# Endovascular Treatment of Ruptured Blood Blister-like Aneurysms Using the LVIS EVO Stents

**DOI:** 10.3390/jcm12031089

**Published:** 2023-01-30

**Authors:** Kinga Kubiak, Wojciech Poncyljusz

**Affiliations:** Department of Diagnostic Imaging and Interventional Radiology, Pomeranian Medical University, 71-252 Szczecin, Poland

**Keywords:** blood blister-like aneurysm, embolization, endovascular treatment

## Abstract

Blood blister-like aneurysms (BBAs) are rare cerebrovascular lesions that face serious challenges in surgical as well as endovascular treatment. In this paper, we present our experience in treating BBAs using the LVIS EVO stents. A total of 10 patients (mean age of 56.1 years) with 13 BBAs, who were admitted to our university hospital between April 2020 and November 2021 with a subarachnoid hemorrhage (SAH) due to aneurysm rupture, were treated using the LVIS EVO stents. Treatment of the BBAs consisted of stent-assisted coiling in four patients and stenting in six patients. The aneurysms were located within ICA (84.6%), VA (7.7%), and MCA (7.7%). Placement of the LVIS EVO stents was successful in all patients. No technical complications were observed. One in-stent thrombotic event occurred during the procedure. MRA for one-year follow-up was performed in nine patients. One patient died (Hunt and Hess Grade IV). LVIS EVO stents may be a beneficial treatment option for BBAs, as they provide high occlusion rates. However, the long-term efficacy remains uncertain.

## 1. Introduction

Blood blister-like aneurysms (BBAs) are a rare cerebrovascular pathology accounting for approximately 0.3–1% of all intracranial aneurysms [1,2,3] and 0.5–2% of all ruptured aneurysms [4,5]. Although such lesions can occur in various locations [2,6], they originate most frequently in non-branching sites of the ICA [2,7].

BBAs are characterized by a small size (<3 mm), thin and fragile walls, the absence of an identifiable neck, nonbranching origin, rapid growth, an increased risk of rupture, and a poorer prognosis comparing to saccular aneurysms [2]. The average age of the affected population tends to be lower than that of patients with saccular aneurysm [8]. A higher prevalence of BBAs is observed in females, right-sided ICA, and patients with hypertension [8,9].

The most prevalent clinical presentation of BBAs is an acute subarachnoid hemorrhage. Due to the small size, they are frequently missed during the initial computed tomography angiogram (CTA) or the initial digital subtraction angiography (DSA). Special consideration must be given to the radiological evolution of BBAs following rupture, as their transition to a saccular shape might be observed several days after bleeding.

Nonetheless, the key points include not only difficulties in their diagnosis, but also the complexity in treatment, which remains challenging. The management of BBAs is demanding and hazardous due to their exceptionally thin wall. These aneurysms, which are particularly prone to intraoperative rupture, may pose significant difficulties for both microsurgical and endovascular approaches. In recent years, endovascular treatment has emerged as the primary option for the treatment of blood blister-like aneurysms owing to the advancement of endovascular treatment techniques and equipment [10]. It has been reported that the endovascular treatment of blood blister-like aneurysms is linked to lower rates of morbidity and mortality, and better outcomes than surgery [2].

One of the latest microstents for cerebral vasculature is the self-expanding LVIS EVO (MicroVention, Terumo, Aliso Viejo, CA, USA). This stent comprises sixteen nitinol wires with a platinum core. The wires are braided using drawn filled tube (DFT) technology, which makes them visible under fluoroscopy. There are four radiopaque markers located on each end of the stent. The stents have short and flared ends (0.5 mm), which provide adequate anchorage, especially for small and tortuous vessels. LVIS EVO stents are available in a wide variety of lengths (range from 12 mm to 34 mm), for vessels from 2 mm to 4 mm. Shorter flared ends, improved visibility, and smaller cell size are some of the potential improvements compared to other braided stents, such as LVIS and LVIS Jr. (MicroVention, Terumo, Aliso Viejo, CA, USA). LVIS EVO stents have a high metal coverage of up to 28%, depending on their size and the parent vessel’s diameter. Due to the partial flow-diverting effect generated by the device, the intra-aneurysmal flow can be reduced [11].

## 2. Materials and Methods

This was a retrospective, single-center study of prospectively gathered data that was approved by the local institutional review board (Bioethics Committee no. KB-0012/29/05/2020/Z). The research included patients with ruptured blood blister-like aneurysms treated endovascularly between April 2020 and November 2021. The median age of the patients was 56.1 years (range 38–71 years). The analyses were conducted on the basis of demographic information, clinical data, aneurysm location and morphology, technical outcomes, and complication rate. MRA for one-year follow-up was performed in 9 patients. One patient died (Hunt and Hess Grade IV) in the ICU due to SAH complications. The aneurysms’ size and morphology were examined using the pre-procedural angiograms.

All endovascular procedures were performed under general anesthesia. Using the Seldinger technique, a 6F sheath introducer (Cordis, Bridgewater, NJ, USA) was inserted into the right femoral artery. A bolus of 1000 units of heparin was injected via the femoral sheath. The flush systems utilized for microcatheters contained 500 units of heparin per liter.

In order to determine the morphology of the lesions and the local vascular anatomy, a pre-treatment digital subtraction angiography (DSA) and rotational angiography with 3D reconstruction were performed (Philips Azurion Clarity IQ-Medical Systems Nederland BV). Stent selection was based on precise measurements of the proximal and distal artery diameters. A guiding catheter, Chaperon 6F (MicroVention, Terumo, Aliso Viejo, CA, USA), was placed into the C1 segment of the internal carotid artery or the V1 segment of vertebral artery. The Headway 17 microcatheter (MicroVention, Terumo, Aliso Viejo, CA, USA) was advanced over a 0.014-inch Traxcess microguidewire (MicroVention, Terumo, Aliso Viejo, CA, USA) and positioned distally to the aneurysm. Subsequently, the LVIS EVO stent was deployed into the parent artery, covering the aneurysm’s neck. In 4 cases, in which the treatment consisted of stent-assisted coiling, the jailing technique was used. After implantation of the stent and platinum coils, DSA and Vaso-CT were performed to determine the final stent position. The vascular closure system—FemoSeal (70%) or AngioSeal (30%) (Terumo Corporation, Tokyo, Japan)—was used to close the femoral artery puncture site.

Two hours prior to the treatment, 7 of 10 patients received a loading dose of 600 mg of clopidogrel and 300 mg of acetylsalicylic acid. The rest of the patients (3/10) received 180 mg of ticagrelor and 300 mg of acetylsalicylic acid. Patients were maintained on dual antiplatelet therapy (acetylsalicylic acid 150 mg, clopidogrel 75 mg) for three months following the embolization.

## 3. Results

**Figure 1:**
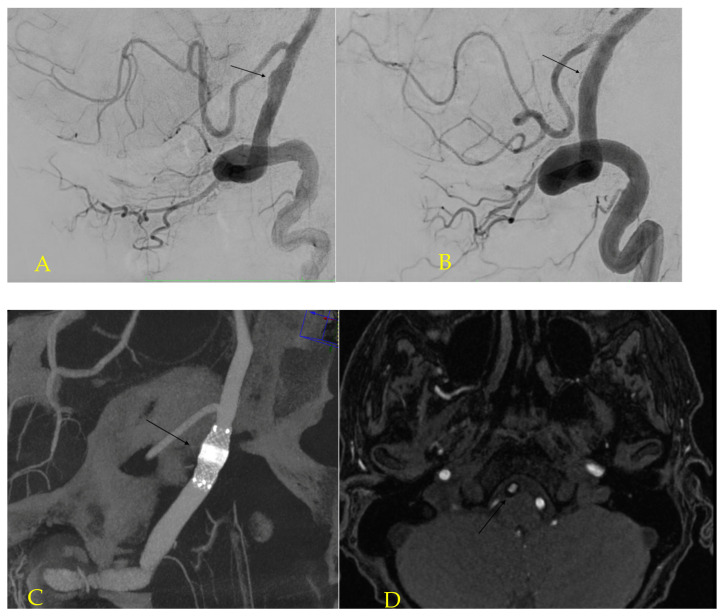
Pretreatment right vertebral lateral angiography (**A**) shows the blood blister-like aneurysm (arrow) below posterior inferior cerebellar artery (PICA). Postembolization angiography (**B**) reveals reduced filling of the aneurysm’s sac. Excellent apposition to the vessel wall and compacting the mesh around the aneurysm’s neck are visualized on intraprocedural contrast-enhanced flat panel detector CT (MIP reconstruction) (**C**). One year following the treatment, MRI revealed good positioning of the stent (arrow) and complete occlusion of the aneurysm (**D**).

Between April 2020 and November 2021, a total of 10 patients were treated with LVIS EVO stents due to blister-like aneurysms rupture. Table 1 summarizes the patients’ characteristics. There was no discernible difference in sex among the patients (five men, five women). The group comprised four patients with hypertension, one with diabetes, and one smoker. Regarding location, 11 of 13 (84.6%) BBAs had a typical location (nonbranching site of ICA): one was found in MCA (7.7%) and one in VA (7.7%) (Figure 1). At the time of treatment, the mean BBAs size was 1.85 mm (range 1–2 mm). Three patients had multiple aneurysms, which were located close enough for a single stent treatment. Five out of thirteen aneurysms were initially identified on CTA, seven aneurysms were found during the following DSA, and one was detected during the DSA performed 7–10 days after SAH.

**Figure 2:**
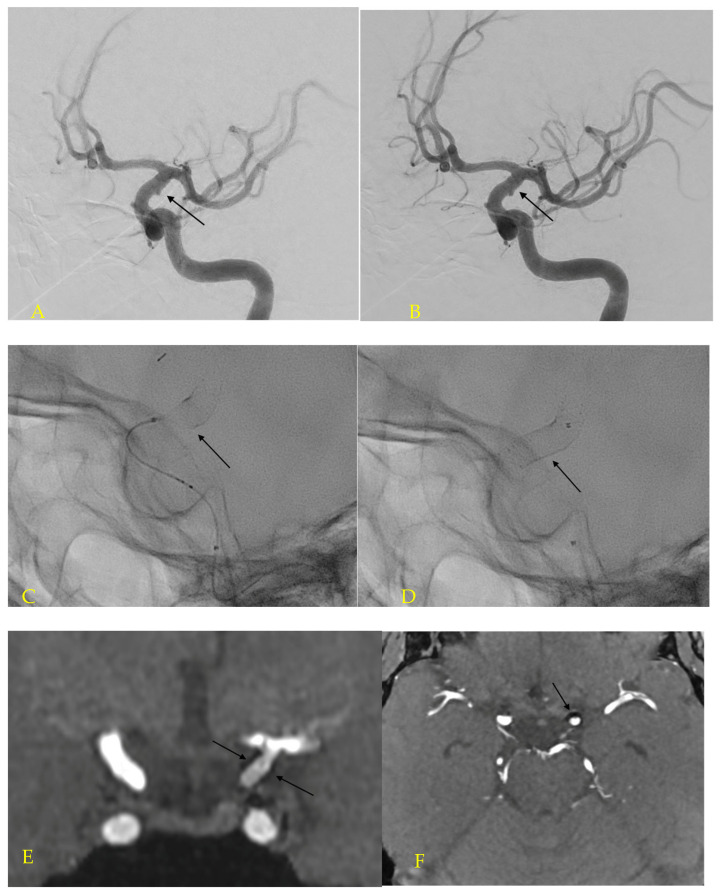
An oblique view of a left carotid artery angiogram revealed a tiny aneurysmal bulge in the supraclinoid segment of ICA (**A**), treated with the stent only and the post-procedural angiogram demonstrated filling the aneurysm (**B**). Fluoroscopic view during the stent positioning and implantation (**C**), and post stent deployment with the microcatheter markers in the parent vessel (**D**). Coronal (**E**) and axial (**F**) views of the 1-year MR TOF follow-up shows full occlusion of the treated aneurysm and good visualization of stent wall (arrows).

There were no device-related complications. All of the stents were successfully delivered and positioned in the desired location (Figure 2). Vaso-CT was performed to evaluate the stents’ shape and apposition to the vessel wall. In one patient, transient in-stent thrombosis occurred, most likely due to clopidogrel resistance. It was successfully treated with eptifibatide (intravenous bolus of 10 mL 2 mg/mL, and then intravenous infusion of 100 mL 0.75 mg/mL, according to the weight). We assume that the instantaneous reaction after the i.v. administration of eptifibatide was evidence of antiplatelet therapy resistance. In this case, we immediately switched clopidogrel to ticagrelor. At the end of the procedure, the control DSA and Vaso-CT were performed. In all cases treated with the stent and coils, the angiograms showed complete occlusion, and partial occlusion in some patients treated with the stent only. The follow-up MRI performed one year after the procedure showed complete occlusion of all of the aneurysms.

## 4. Discussion

Blood blister-like aneurysms were first characterized in 1969 by Sundt and Murphy as unusual sessile aneurysms of the supraclinoid segment of the ICA [12]. In 1992, the term “blood blister—like” was introduced to the English language literature by Shigeta [13].

The pathophysiology of BBAs remains obscure. Histologically, BBAs are characterized by the presence of a focal wall defect with a gap in the internal elastic lamina and media, covered with a thin adventitia as well as a fibrous tissue [14,15]. According to the literature, atherosclerosis and hemodynamic stress are factors that play a major role in the development of BBAs [9]. Furthermore, it has been reported that 40–89% of cases are associated with arterial dissection [15].

The diagnosis of BBAs is routinely based on the anatomic, morphologic, and clinical aspects of the condition. Although CT angiography (CTA) has been found to have high specificity and sensitivity in detecting ruptured aneurysms, its value in the context of BBAs has rarely been evaluated [16]. BBAs are frequently undiagnosed at admission, especially due to their small size and broad-based shape, which makes the diagnosis difficult. As a result, DSA after CTA is frequently required. Gaughen et al., described a series of six patients with BBAs of the ICA who were evaluated with CTA before DSA [17]. Four out of a total of six BBAs were detected by the CTA, resulting in a 33% false negative rate. In our study, 8/13 (61.5%) patients required DSA after CTA, and 1/13 (7.69%) BBA was detected at the second DSA control, 7 days after the bleeding. Therefore, DSA is still widely used as the gold standard for identifying BBAs. Anteroposterior and lateral angiographic images will not optimally display these lesions because they typically originate from the anterior wall of the supraclinoid segment of ICA [18]. Our experiences showed that except for the three obligatory views, an additional contralateral oblique view should be performed in each patient. This way, we were able to find four aneurysms, which were not visible on typical views. When a BBA is suspected, rotational three-dimensional angiography is especially useful [18,19]. Three-dimensional DSA was very helpful in analyzing and confirming the presence of BBAs in our group of patients before the embolization procedure. When the initial angiography is inconclusive and appears to be negative, it is advisable that a close follow-up should be performed, as rapid transitions from small suspicious bulges to saccular-type lesions have been described [18].

A variety of endovascular techniques, including stenting, coiling, stent-assisted coiling, and flow-diverting stents, have been applied for BBAs treatment. However, a standardized treatment protocol has not yet been established. Ruptured BBAs have a very fragile neck and a shallow sac, making simple coiling difficult or even impossible to perform, and they require stent-assisted coil embolization [20]. Furthermore, the stent can provide a bridge for vascular endothelial cells to cover the BBA’s neck [21].

LVIS EVO is a novel, self-expandable braided stent with drawn filled tube technology (DFT), which improves the visibility of the wire and facilitates stent deployment. In terms of its function and flexibility, it is similar to Accero (Acandis, Pforzheim, Germany) [22] and Leo stents (Balt, Montmorence, France) [23]. LVIS EVO appears to be more flexible and very well adapted to vascular anatomy. In our study, all stents were deployed in the desired location without any procedure-related complications. However, a shortening of the proximal part should be considered during the implantation, similarly to some flow-diverters.

In our group of patients, SAC using the jailing technique was performed in four cases. In the other six cases, only stenting was performed, as coil implantation was impossible.

The LVIS EVO stent has a high metal coverage of up to 28%, which makes it equivalent to the minimum metal coverage of flow-diverting stents. A standard flow-divert stent has a somewhat higher metal coverage surface area, up to 30–35%.

Endovascular techniques may result in aneurysm regrowth and rebleeding. In the case of stent-assisted coiling, there is still a possibility of coils implantation or inserting a second stent using the stent-within-a-stent technique. Flow-diverting stents have a higher tendency to stimulate platelet aggregation (thrombogenicity) compared to braided stents.

There are no reports of the treatment of BBAs with LVIS EVO stents, but they seem to be a good alternative for BBAs management. Several studies have demonstrated the safety and efficacy of this treatment method for saccular aneurysms [11,24]. We present our series of patients successfully treated with this new device. This study has several limitations. Despite the limited experience with the device, it can be stated that some issues, such as BBAs regrowth or in-stent thrombosis, cannot be eliminated due to two factors: first, the small sample size, and second, the lack of an extended follow-up period.

## 5. Conclusions

Our research shows cases of ruptured BBAs that were successfully treated with LVIS EVO stents. However, further studies with a large population of patients are required in order to fully evaluate the effectiveness of the treatment.

## Figures and Tables

**Table 1:** The patients’ characteristics.

Case No	Sex/Age	Localization	Multiple Aneurysms	H&H Grade	Hypertension	Diabetic	Smoker
1.	M/48	LICA		2			
2.	K/71	LICA		3	+		
3.	K/55	LICA		3			
4.	M/59	LICA	+	2	+		
5.	K/54	RVA	+	2			
6.	K/55	RICA		4		+	
7.	M/50	LICA		1			
8.	K/69	RICA		3	+		+
9.	M/38	MCA	+	2			
10.	M/62	RICA		2	+		

Internal carotid artery (ICA), middle cerebral artery (MCA), vertebral artery (VA).

## Data Availability

Not applicable.
